# Integration of CdSe/CdSe_*x*_Te_1−*x*_ Type-II Heterojunction Nanorods into Hierarchically Porous TiO_2_ Electrode for Efficient Solar Energy Conversion

**DOI:** 10.1038/srep17472

**Published:** 2015-12-07

**Authors:** Sangheon Lee, Joseph C. Flanagan, Joonhyeon Kang, Jinhyun Kim, Moonsub Shim, Byungwoo Park

**Affiliations:** 1 WCU Hybrid Materials Program, Department of Materials Science and Engineering, Research Institute of Advanced Materials, Seoul National University, Seoul 08826, Korea; 2 Department of Materials Science and Engineering, University of Illinois at Urbana-Champaign, Urbana, Illinois 61801, United States

## Abstract

Semiconductor sensitized solar cells, a promising candidate for next-generation photovoltaics, have seen notable progress using 0-D quantum dots as light harvesting materials. Integration of higher-dimensional nanostructures and their multi-composition variants into sensitized solar cells is, however, still not fully investigated despite their unique features potentially beneficial for improving performance. Herein, CdSe/CdSe_*x*_Te_1−*x*_ type-II heterojunction nanorods are utilized as novel light harvesters for sensitized solar cells for the first time. The CdSe/CdSe_*x*_Te_1−*x*_ heterojunction-nanorod sensitized solar cell exhibits ~33% improvement in the power conversion efficiency compared to its single-component counterpart, resulting from superior optoelectronic properties of the type-II heterostructure and 1-octanethiol ligands aiding facile electron extraction at the heterojunction nanorod-TiO_2_ interface. Additional ~32% enhancement in power conversion efficiency is achieved by introducing percolation channels of large pores in the mesoporous TiO_2_ electrode, which allow 1-D sensitizers to infiltrate the entire depth of electrode. These strategies combined together lead to 3.02% power conversion efficiency, which is one of the highest values among sensitized solar cells utilizing 1-D nanostructures as sensitizer materials.

Semiconductor sensitized solar cells (SSCs) are promising as one of the next generation photovoltaics (PVs) due to the attractive optoelectronic properties of semiconductor light harvesters. In addition to the high absorption coefficient, bandgap and band edge positions can be tuned by the quantum confinement effect and composition^1^,^2^,^3^,^4^,^5^,^6^. There is also the possibility of multiple exciton generation, which may lead to the PVs overcoming Shockley-Queisser limit^7^,^8^,^9^. Two main approaches exist for assembling light-harvesting semiconductor sensitizers on mesoporous metal oxide electrodes such as TiO_2_ (mp-TiO_2_). One approach is the *in situ* route, where the semiconductor sensitizers are grown directly on the surface of mp-TiO_2_ electrodes^10^,^11^. Successive ionic layer adsorption and reaction (SILAR) and chemical bath deposition (CBD) are typical methods belonging to this category^12^,^13^,^14^,^15^. The *in situ* route benefits from the intimate contact between the sensitizer and the TiO_2_, but often suffers from the complication of sensitizer size/shape control and relatively poor crystallinity of the assembled sensitizers^16^,^17^. On the other hand, in the *ex situ* route, semiconductor sensitizers are synthesized prior to the assembly onto the mp-TiO_2_ electrode, and sensitization is carried out thereafter via direct adsorption, linker-assisted adsorption, electrophoretic deposition (EPD), or similar approaches^18^,^19^,^20^,^21^. In the *ex situ* assembly route, pre-synthesized sensitizers bear insulating ligand molecules on their surfaces, making the contact between the sensitizers and the mp-TiO_2_ electrode less intimate^22^,^23^. However, SSCs derived from an *ex situ* assembly route can benefit from high-quality sensitizers with well-defined size/shape and high crystallinity, and over 8% certified power conversion efficiency (PCE) has been achieved recently with quantum dots (QDs)^24^,^25^.

Despite the versatility of the *ex situ* assembly route allowing separately prepared sensitizers with various shapes, sizes, and compositions, most efforts have been focused solely on the 0-D semiconductor QDs as sensitizer materials^18^,^19^,^20^,^21^,^22^,^23^,^24^,^25^. One-dimensional nanorods have been known to have fascinating optoelectronic properties different from QDs, which are potentially conducive to realizing better-performance SSCs. Compared to the QDs in which photogenerated electron-hole pairs are strongly bound by electrostatic forces^26^, 1-D nanorods are expected to have weaker binding for the electron-hole pairs in their elongated structure^27^. This implies that the electron-hole recombination, one of the major factors deteriorating the performance of SSCs, will be less significant in the 1-D sensitizers than QDs. Therefore, 1-D nanorods can be a decent alternative or complement for the conventional SSCs utilizing QD sensitizers. However, 1-D nanostructures and their multi-composition variants have been given only a limited attention in this field up to now^28^,^29^. This is because of two major complications of integrating those nanostructures into SSCs: one is the difficulty of synthesizing well-defined 1-D sensitizer itself with proper composition and nanoscale morphology guaranteeing the optimal photovoltaic performance, and the other is the spatial incompatibility of the long 1-D sensitizers with the conventionally available nanoporous photoelectrodes derived from metal-oxide nanoparticles, by which the full penetration of 1-D sensitizers through the photoelectrode is impeded. Various strategies to improve PCE are also remaining unexplored in the case of 1-D nanostructure-sensitized PVs, such as ligand-exchange, transition-metal doping, formation of core-shell structures, and other surface-passivation technologies^30^,^31^,^32^,^33^.

Herein, we report for the first time the CdSe/CdSe_*x*_Te_1−*x*_ type-II heterojunction nanorods (HNRs) as light harvesting sensitizers for SSCs. About 40% enhancement of the PCE is achieved using HNRs compared to the PCE using CdSe nanorods (NRs), which can be attributed to the inherent efficient charge separation across the type-II heterointerface and favorable effects of 1-octanethiol (OT) surface ligands on the TiO_2_-HNR interfacial charge transfer. Furthermore, to circumvent the spatial incompatibility of long 1-D sensitizers with conventional mp-TiO_2_ electrodes, polystyrene (PS) microbeads are added as sacrificial additives to render large percolating pores in the mp-TiO_2_ and thereby facilitate the infiltration of HNRs throughout the entire electrode depth. The CdSe/CdSe_*x*_Te_1−*x*_ HNR-SSC with pore-engineered electrode is shown to reach 3.02% PCE, which is one of the highest values among SSCs using the 1-D sensitizers.

## Results

### Optical Properties of NRs and HNRs

**Figure 1:**
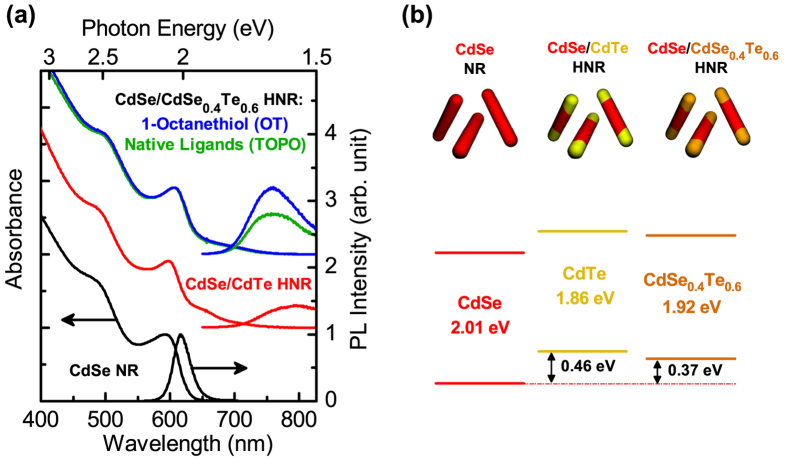
(**a**) Absorption and photoluminescence (PL) spectra of CdSe NRs, CdSe/CdTe HNRs, and CdSe/CdSe_0.4_Te_0.6_ HNRs with both native ligands (TOPO) and 1-octanethiol ligands (OT). The PL spectra for CdSe NRs and 1-octanethiol-capped CdSe/CdSe_0.4_Te_0.6_ HNRs are normalized to the absorbance at the CdSe first exciton peak. The PL spectra for native ligand capped CdSe/CdSe_0.4_Te_0.6_ and CdSe/CdTe HNRs are normalized to the absorbance at the excitation then scaled relative to the PL spectrum of the 1-octanethiol capped CdSe/CdSe_0.4_Te_0.6_ HNRs. (**b**) Schematic illustration of different 1-D sensitizers and energy-level diagram of each component consisting of type-II HNRs based on previous reports^34^,^35^.

**Figure 2:**
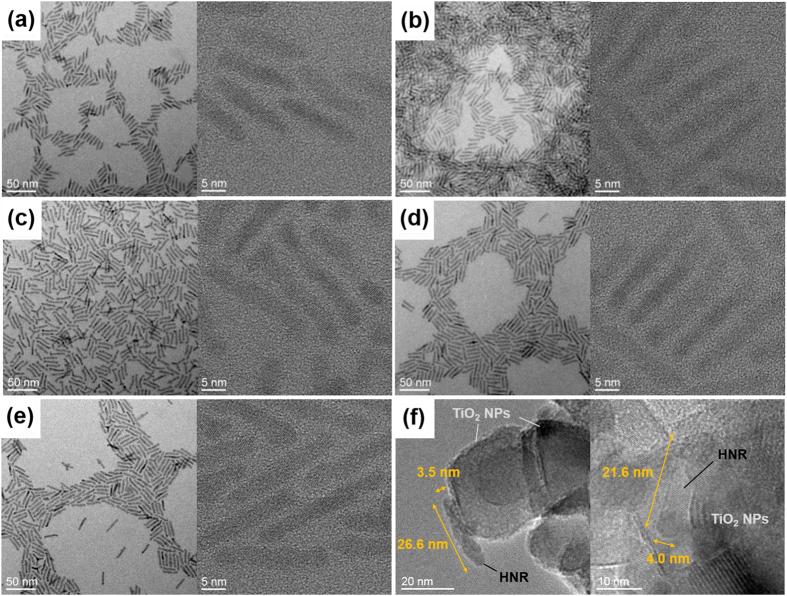
Transmission electron micrographs of (**a**) the seed CdSe NRs, (**b**) TOPO-CdSe NRs, (**c**) TOPO-CdSe/CdTe HNRs, (**d**) TOPO-CdSe/CdSe_0.4_Te_0.6_ HNRs, (**e**) OT-CdSe/CdSe_0.4_Te_0.6_ HNRs, and (**f**) OT-CdSe/CdSe_0.4_Te_0.6_ HNRs anchored to the TiO_2_ nanoparticles.

Absorption and photoluminescence (PL) spectra of NRs and HNRs used in this study are shown in Fig. 1. Each sample is synthesized from CdSe NR seeds, with the second components grown at the tips. TEM images of NRs and HNRs are presented in Fig. 2, showing that HNRs synthesized from ~15 nm long CdSe NR seeds are ~25 nm long in average. The absorption spectrum for CdSe-only NRs shows a peak near 600 nm with a sharp PL peak at 615 nm. When CdTe is introduced as the second component, an absorption shoulder appears at 650 nm due to the smaller bandgap of CdTe. The absorption tail extends beyond 700 nm as a result of the charge transfer transition from the CdTe valence band to the CdSe conduction band. Recombination across the CdSe/CdTe interface occurs at energies lower than the bandgap of either component, shifting the PL peak to ~800 nm and broadening it considerably. Alloyed CdSe_0.4_Te_0.6_ also forms a type-II junction with CdSe, therefore many of the absorption and PL features for these HNRs are similar. The difference between the PL peak position of the HNRs and that of the CdSe seeds, measured to be 0.37 eV, was used to determine the concentration of Te in the alloy following the calibration introduced in our previous work^34^,^35^. The CdSe/CdSe_0.4_Te_0.6_ HNRs recapped with 1-octanethiol retain the absorption features seen with the native ligands, but have higher PL intensity and better stability in air, similar to the 1-octanethiol capped CdSe/CdTe HNRs we reported recently^36^. For every heterostructured sample, the absorption spectrum extends beyond 700 nm, and should allow a greater portion of the incident solar spectrum to be collected in a photovoltaic device compared to single-component CdSe.

### Photovoltaic Properties of NR- and HNR-Sensitized PV Devices

**Figure 3:**
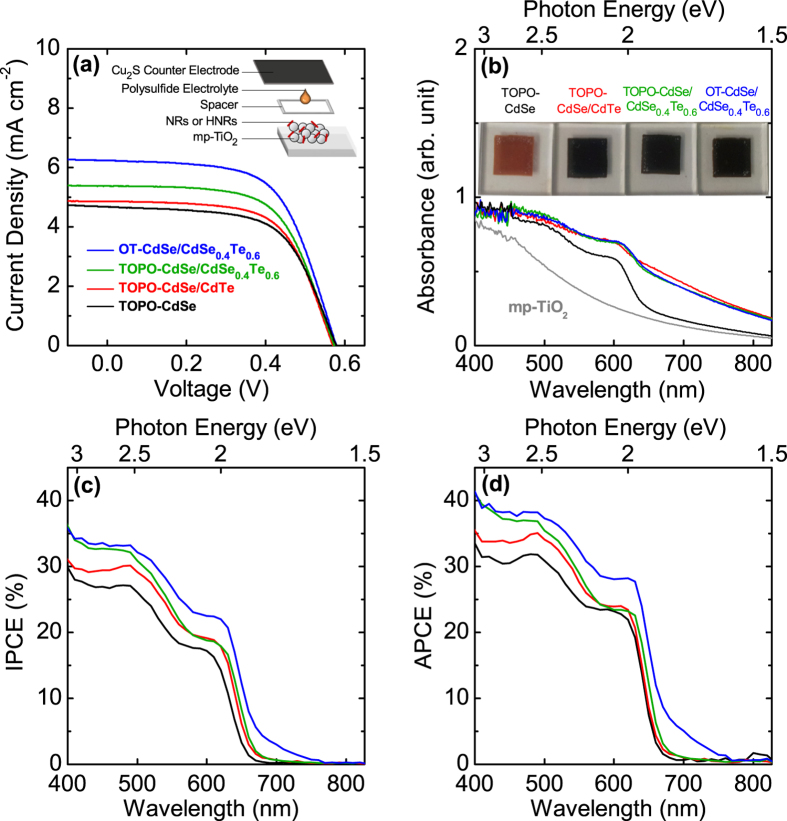
(**a**) Photocurrent-voltage curves (averaged line from two sets of samples prepared in parallel), (**b**) absorption spectra of the sensitized films, (**c**) incident photon-to-current conversion efficiency (IPCE), and (**d**) absorbed photon-to-current conversion efficiency (APCE) of NR- and HNR-sensitized solar cells with different sensitizer structures, compositions, and surface ligands (native ligands (TOPO) or 1-octanethiol (OT)).

Photocurrent-voltage (*J*-*V*) characteristics, absorbance spectra of sensitized films, incident photon-to-current efficiency (IPCE), and absorbed photon-to-current efficiency (APCE) spectra for various NR- and HNR-sensitized PV devices are presented in Fig. 3. Photovoltaic parameters of the devices are given in Table 1. Every device exhibits similar open-circuit voltages (*V*_*oc*_) and fill factors (*FF*), but short-circuit current density (*J*_*sc*_) values show remarkable differences. As the time constant of the electron transfer from CdTe (or CdSe_0.4_Te_0.6_) tips to CdSe in HNRs (<400 fs) is much shorter than that of the electron transfer from CdTe to TiO_2_ (~1 ns)^34^,^36^,^37^, we believe that most of the photocurrent would come from the electron transfer at the CdSe-TiO_2_ interface (for CdSe NRs and HNRs). If a major portion of HNRs are anchored to TiO_2_ only by their tips, electron extraction at the CdTe/TiO_2_ interface should compete with more efficient electron transfer to CdSe. Moreover, electron transfer from CdSe to CdTe is not favored considering the band diagram in Fig. 1b, leading to poor fill factor as we previously reported in the case of organic-inorganic hybrid solar-cell structure utilizing curved CdSe/CdTe HNRs as inorganic light harvesters^38^. Such a loss of fill factor was not observed in HNR-sensitized PV devices, and therefore we believe that most of HNRs incorporated in the mp-TiO_2_ frame have some portion of CdSe directly anchored to the TiO_2_ nanoparticles. The TEM images of HNRs anchored on mp-TiO_2_ in Fig. 2f partially support this argument.

The TOPO-CdSe/CdTe HNR device delivers slightly higher *J*_*sc*_ compared to the TOPO-CdSe device. Considering that the photocurrent generated from single-component CdTe sensitizers is generally far lower than the single-component CdSe sensitizers presumably due to the recombination loss from the charge-carrier trapping^38^,^39^,^40^,^41^, higher *J*_*sc*_ of the TOPO-CdSe/CdTe HNRs than that of the TOPO-CdSe NRs (with the same dimension of ~25 nm) cannot be explained simply by summing up the photocurrent generated from individual CdSe and CdTe components in HNRs. Due to the unique feature of type-II band offset in HNRs, where photoexcited electron-hole pairs are innately separated, the electron extraction process at the TiO_2_-sensitizer interface becomes more efficient with HNRs than with the single-component NRs^39^. This explains the increase of IPCE over the entire wavelength region for the TOPO-CdSe/CdTe HNR-SSCs compared to the case of TOPO-CdSe single-component NRs working as light harvesters. Furthermore, the type-II interface of TOPO-CdSe/CdTe HNR leads to a charge-separated state (CSS) absorption^39^, enabling the utilization of less energetic photons close to the near-infrared region. The effect of CSS absorption inherent for the HNR sensitizers is reflected in the extended absorption tail of the sensitized films (Fig. 3b) compared to that of the TOPO-CdSe NR device.

In terms of the performance of TOPO-CdSe/CdTe HNR device, the stability of CdTe component in polysulfide electrolyte is a major concern, and therefore should be discussed. It has been well known that in the case of CdTe-sensitized SSCs using polysulfide electrolyte, anodic corrosion due to the ineffective hole scavenging from CdTe leads to the degradation of CdTe and low photovoltaic performance^42^,^43^. This degradation can be lessened by forming a semiconductor shell preventing CdTe cores from directly facing the polysulfide electrolyte^25^. Even though the CdTe component of the CdSe/CdTe HNRs does not have such a core-shell structure, our devices are finally treated to have ZnS passivation layer and therefore CdSe/CdTe HNR devices are not much affected by the anodic corrosion and exhibit reasonable performance.

In the case of TOPO-CdSe/CdSe_0.4_Te_0.6_ HNR device, ~11% extra enhancement of *J*_*sc*_ compared to that of the TOPO-CdSe/CdTe NR device is observed. CdSe/CdSe_0.4_Te_0.6_ HNRs allow faster charge separation compared to the CdSe/CdTe HNRs and have lower valence band position in the alloyed tip^34^, which might lead to the *J*_*sc*_ improvement by aiding facile charge extraction to TiO_2_. Furthermore, in the case of HNRs with alloyed tips, enhanced chemical stability of alloyed tips is also thought to contribute to achieve the improved photocurrent^39^.

A noticeable further enhancement in the photocurrent density is observed by exchanging the surface ligands on the CdSe/CdSe_0.4_Te_0.6_ HNRs, from native TOPO, ODPA, and TOP (simply referred to here as TOPO) ligands to 1-octanethiol (OT). As the absorption spectra obtained from mp-TiO_2_ films sensitized with TOPO-CdSe/CdSe_0.4_Te_0.6_ and OT-CdSe/CdSe_0.4_Te_0.6_ are quite similar (Fig. 3b), the *J*_*sc*_ upsurge may be attributed to the enhancement of electron injection/collection efficiency rather than the difference in the light harvesting from both sensitizers. Such an interpretation is further supported by the trend shown in the APCE spectra (Fig. 3d): APCE is represented as the product of charge collection efficiency (*η*_*col*_) and charge injection efficiency (*η*_*inj*_), which can be calculated from the following relation (equation (1))^44^:


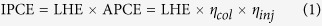


where the term LHE represents light harvesting efficiency. By comparing Fig. 3c,d, it is found that the APCE onset of the OT-CdSe/CdSe_0.4_Te_0.6_ device is extended to ~760 nm compared to that of the TOPO-capped HNR devices. This result is in line with the previous report of the photocurrent spectra extended over ~700 nm for the 1-octanethiol capped CdSe/CdTe HNRs^36^. Moreover, the difference of IPCE among the TOPO-capped sensitizer devices almost vanishes from the ~580-nm wavelength in the APCE spectra, showing that the charge collection and injection efficiency of the TOPO-capped samples are quite similar in this wavelength region. However, APCE near ~480 nm corresponding to the *X*_2_ excitonic band of CdSe still shows composition dependence, which we might attribute to the aforementioned effect from the faster charge-separation kinetics of type-II band offset and higher photocurrent obtainable from alloyed tips than CdTe tips. However, clear interpretation for this phenomenon is not established yet and further study is needed.

It is readily deduced that *η*_*col*_ can be improved from the 1-octanethiol capping, as it has been well known that the thiol recapping of CdTe reduces the surface defect sites responsible for any unwanted recombination of photo-generated charge carriers^36^,^40^,^41^. This feature is also found in Fig. 1a showing higher PL intensity of OT-CdSe/CdSe_0.4_Te_0.6_ than TOPO-CdSe/CdSe_0.4_Te_0.6_, which is presumably resulted from the suppressed recombination by 1-octanethiol capping. Improvement of carrier collection from enhanced electric-field throughout the HNR absorber layer is another possible factor leading to the improved *J*_*sc*_, but it may hardly be the case in our devices considering photocurrent saturation under the reverse-biased condition in the *J*-*V* curve (Fig. 3a) and our sensitized solar cell structure utilizing monolayer HNRs as light absorber layer^45^.

**Figure 4:**
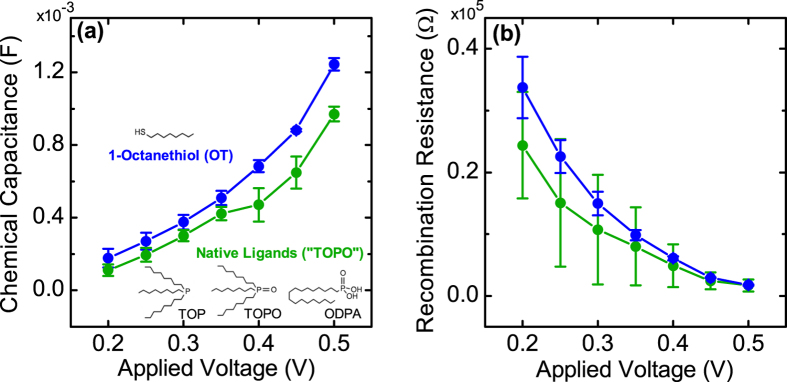
(**a**) Chemical capacitance (*C*_*μ*_) and (**b**) recombination resistance (*R*_*rec*_) of TiO_2_ photoelectrodes sensitized with CdSe/CdSe_0.4_Te_0.6_ HNRs having different passivation ligands, measured by electrochemical impedance spectroscopy (EIS) under dark conditions. Each point is the average value from two sets of samples prepared in parallel.

However, the difference of *η*_*inj*_ from 1-octanethiol recapping is not self-evident and further investigation on the TiO_2_-OT-HNR interface is needed. To elucidate the interface properties, electrochemical impedance spectroscopy (EIS) was carried out^46^,^47^. The mp-TiO_2_ electrodes sensitized with TOPO-capped CdSe/CdSe_0.4_Te_0.6_ HNR and OT-capped CdSe/CdSe_0.4_Te_0.6_ HNR sensitizers were chosen for the EIS measurement, to examine the capping ligand effect by excluding any side effects from the HNR composition and different loading amount of HNRs on mp-TiO_2_ electrodes. Figure 4 presents the chemical capacitance (*C*_*μ*_) and recombination resistance (*R*_*rec*_) extracted from the measured impedance spectra for both electrodes, exhibiting that the electrode sensitized with OT-CdSe/CdSe_0.4_Te_0.6_ HNR shows higher chemical capacitance in the measured voltage range and higher recombination resistance in average. Higher chemical capacitance is generally interpreted as evidence for the downshift of the TiO_2_ conduction-band minimum (CBM)^48^,^49^. However, in our case, we believe it is the upshift of the CdSe CBM of CdSe/CdSe_0.4_Te_0.6_ HNR with OT, as reported for CdSe nanocrystals^50^,^51^, that may be responsible for the increased chemical capacitance. Furthermore, TiO_2_ in both devices have been treated with 3-MPA, and therefore the effects of 3-MPA on the CBM of TiO_2_ is not likely to be very different. Whether it is the downshift of the TiO_2_ CBM or the upshift of the CdSe CBM, both cases lead to a larger offset between TiO_2_ CBM and CdSe CBM of CdSe/CdSe_0.4_Te_0.6_ HNR. From the many-state Marcus model describing the electron transfer rate at the metal-oxide and semiconductor-nanoparticle interface^52^, faster electron injection is predicted for this case as the CBM offset serves as the driving force for the electron transfer at the TiO_2_-CdSe interface^53^,^54^,^55^. Less energetic electrons generated in the HNRs may become extractable by this larger injection driving force, which also explains the origin of red-shifted APCE onset for the OT-CdSe/CdSe_0.4_Te_0.6_ device. The higher recombination resistance is believed to result from the reduced recombination by thiol-capping, as explained in the previous section.

Therefore, it is concluded that both *η*_*col*_ and *η*_*inj*_ (equation (1)) are improved through the 1-octanethiol recapping, and the *J*_*sc*_ increase from the OT-CdSe/CdSe_0.4_Te_0.6_ device with the red-shifted IPCE onset compared to the TOPO-CdSe/CdSe_0.4_Te_0.6_ can happen without remarkable increase of the light absorption. The OT-CdSe/CdSe_0.4_Te_0.6_ HNR-SSC shows *J*_*sc*_ of 6.238 ± 0.212 mA cm^−2^ with 2.202 ± 0.131 overall PCE, approximately 33% improvement over the single-component CdSe NR devices with native ligands.

### The Effect of Polystyrene Bead-Induced Percolating Pores on the PV Performance

Even though the power conversion efficiency of the HNR device is much enhanced by utilizing both the type-II HNR sensitizers and ligand exchange, utilization of such 1-D long nanostructures as light harvesters for SSCs is still limited by their spatial incompatibility with the TiO_2_ nanoparticle-based porous photoelectrodes. This type of electrode commonly provides ~20-nm sized pores (BET scale)^56^, which is smaller than the length of these 1-D nanostructures (~25 nm long). However, strategies to circumvent such limitations have been investigated only by a few researchers^28^,^57^.

**Figure 5:**
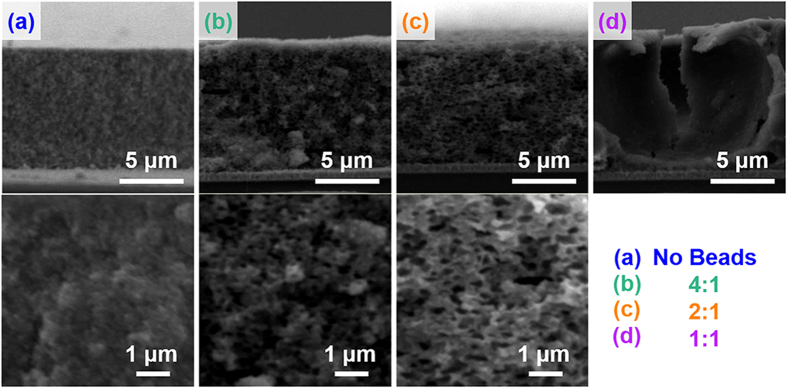
Cross-sectional SEM images of mp-TiO_2_ electrodes derived from the pastes with various PS contents.

**Figure 6:**
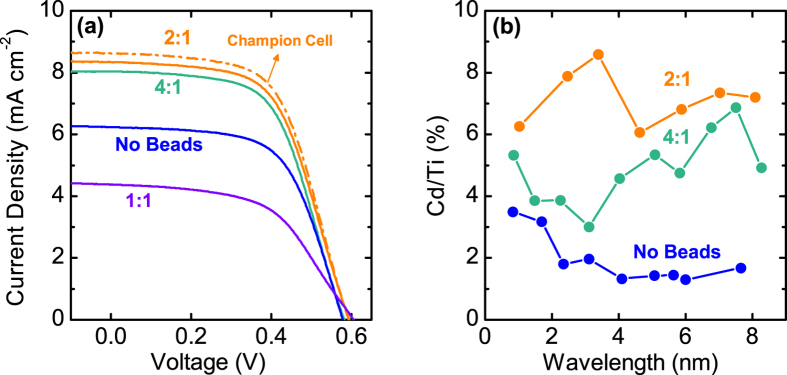
(**a**) Photocurrent-voltage curves (averaged line from two sets of samples prepared in parallel, except for the curve of champion cell) and (**b**) Cd/Ti atomic ratio determined by cross-sectional EDS of OT-CdSe/CdSe_0.4_Te_0.6_ HNR-sensitized electrodes derived from the pastes of various PS contents.

**Figure 7:**
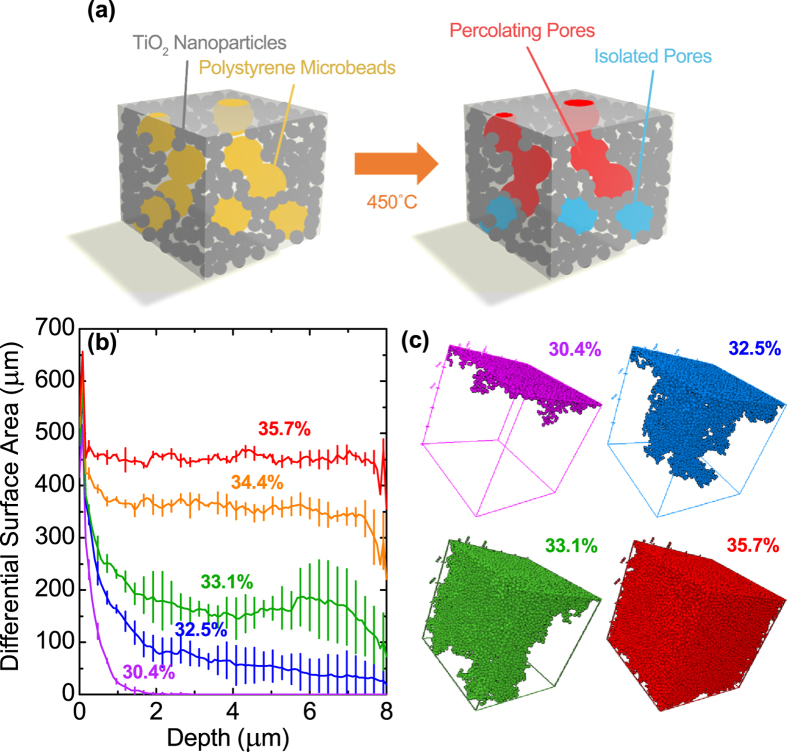
(**a**) Schematic illustration for the formation of percolating large pore channels in mp-TiO_2_ electrode. (**b**,**c**) Monte-Carlo simulation results. (**b**) Differential surface area at the given depth in 8 × 8 × 8 μm^3^ sized continuous cube, calculated from the percolated pores (200 nm in diameter). Each line is averaged from 4 different simulations. (**c**) Visual illustration of typical simulation results for different contents of spherical pores, showing the percolated 200-nm spheres.

To render the internal pore structures of mp-TiO_2_ electrode more suitable for the infiltration of 1-D sensitizers, we utilized polystyrene (PS) microbeads as sacrificial additives for the conventional TiO_2_ paste. Figures 5 and 6b show cross-sectional SEM images and EDS profiles of mp-TiO_2_ electrodes derived from the pastes of different TiO_2_:PS weight ratios. For the electrode without PS beads, Cd/Ti ratio is not homogeneous throughout the electrode cross-section, and abruptly decreases at the ~2-μm depth, suggesting that ~20-nm sized pores in mp-TiO_2_ electrodes are easily clogged by the NR or HNR sensitizers. However, when the PS beads are incorporated into the TiO_2_ pastes and calcined to leave large pores (Fig. 7a), the Cd/Ti ratio at every given depth of the electrode cross-section is highly increased, and the distribution of Cd/Ti values becomes much more homogeneous throughout the entire electrode depth.

Figure 6a shows the *J*-*V* characteristics of HNR devices with different TiO_2_ to PS ratios, the photovoltaic parameters of which are presented in Table 2. Incorporation of PS beads leads to a significant increase in both *J*_*sc*_ and PCE. From these results, we presume that the incorporation of PS beads leads to a more open pore structures, possibly by the percolation of beads to yield large pore channels through which infiltration of HNRs is facilitated. To support this idea, a Monte-Carlo simulation was conducted: 200-nm sized spheres representing PS beads are randomly distributed in an 8 × 8 × 8 μm^3^ space, and the condition of the bead-to-bead percolation allowing the passage of NRHs through the pores induced from adjoining PS beads was estimated from the average length of HNRs (including the capping ligands) and average diameter of TiO_2_ (P25) nanoparticles. The Monte-Carlo simulation results are presented for various volume fraction of PS beads: Fig. 7b exhibits the differential value of internal surface area (d*S*/d*z*) along the depth direction, while Fig. 7c shows 3-D construction of typical simulation results, revealing the percolated 200-nm spheres in the simulation volume.

It is predicted that with ~32.5% volumetric occupation of 200-nm sized PS beads in the TiO_2_ nanoparticle electrode, percolation of beads extends throughout the entire electrode depth (8 μm). Assuming the porosity of mp-TiO_2_ films to be in the range of 0.5–0.6^56^, the PS beads account for the 27.5–32.1% of the total volume in the electrodes with TiO_2_:PS = 4:1 after calcination, which is close to 32.5%. Therefore, the homogeneous Cd/Ti ratio throughout the electrode depth and the improvement of PV performances from the PS-derived hierarchically porous TiO_2_ electrodes can be explained by the percolation of large pores and subsequently facilitated infiltration of HNRs. Furthermore, d*S*/d*z* values increase even further after the formation of electrode-penetrating percolation channel, as seen in Fig. 7b, meaning that the infiltration of HNRs would be more promoted by the addition of more PS beads. Therefore, the Cd/Ti ratio exhibits a further increase for the TiO_2_:PS = 2:1 electrode, and the optimal PV performance is obtained at this ratio. We also believe that this strategy can benefit from the Mie-type light-scattering effects produced by spherical pores filled with electrolytes, as previously explored theoretically (in the case of dye-sensitized solar cells) and experimentally^58^,^59^. However, with a larger amount of PS beads the electrode structure collapses from the aggregation of PS beads forming large voids (Fig. 5d), leading to inferior *J*_sc_ and *FF* due to the loss of active mp-TiO_2_ volume to accommodate HNRs and deteriorated recombination at the direct electrolyte-FTO contact.

Our simple approach utilizing sacrificial spherical additives results in ~32% additional enhancement in *J*_*sc*_ compared to the OT-CdSe/CdSe_0.4_Te_0.6_ HNR-SSC from the mp-TiO_2_ electrode without polystyrene microbead-induced percolating pores, yielding a 3.02% efficient PV device. This is one of the highest values among the SSCs with 1-D sensitizers, and a promising result proving that multi-composition variants of 1-D nanostructures can be fully utilized for this type of photovoltaic device with sensible engineering.

## Discussion

In this work, CdSe/CdSe_*x*_Te_1−*x*_ HNRs were effectively utilized as a light absorber material for semiconductor-sensitized solar cells. Tailored nano-heterostructures and surface passivation with suitable ligand proved to be crucial factors to achieve the enhanced photocurrent density, by utilizing the superior optoelectronic properties of type-II heterostructures and modifying the conduction band level with surface ligands. Furthermore, to circumvent the spatial incompatibility of 1-D sensitizers with the conventional mp-TiO_2_ electrodes, spherical PS bead additives were incorporated to render a hierarchical pore structure in the mp-TiO_2_ electrode. Electrodes with PS-modified pore structures showed highly enhanced PV performance by the formation of large percolating pores inside the electrode, which was also supported by a Monte-Carlo simulation. Consequently, 3.02% efficient HNR-SSC was achieved by integrating the aforementioned approaches, exhibiting that 1- or higher-dimensional nanostructures and their multi-composition variants can potentially be fascinating alternatives as light harvesters for sensitized PVs.

## Methods

### Nanorod Synthesis and Recapping

Trioctylphosphine oxide (TOPO) (90%), trioctylphosphine (TOP) (90%), 1-octanethiol (98.5%), CdO (99.5%), Se powder (99.99%), Te powder (99.997%), and anhydrous toluene were obtained from Sigma-Aldrich. N-octadecylphosphonic acid (ODPA) was obtained from PCI Synthesis. ACS grade chloroform, hexanes, and methanol were obtained from Fisher Scientific. All chemicals were used as received. CdSe NRs, CdSe/CdTe HNRs, and CdSe/CdSe_*x*_Te_1−*x*_ HNRs were synthesized following our previous work with minor differences^34^. In brief, 0.19 g CdO, 1 g ODPA, and 3 g TOPO were added to a 3-neck round-bottom flask and degassed at 150 °C, then heated and stirred at 350 °C for 2 h under argon atmosphere to form the Cd-ODPA complex. The reaction mixture was then cooled to 150 °C and degassed for 10 min. Precursor solutions of 1 M TOP-Se and 1 M TOP-Te were prepared separately in an N_2_-filled glovebox by dissolving elemental Se or Te powder in TOP. Injection solutions were made by diluting the concentrated precursors to the appropriate concentrations with TOP, and 3 mL of 0.33 M TOP-Se was swiftly injected at 320 °C, quenching the reaction mixture to 260 °C. The growth of CdSe nanorods continued for 15 min. Then, the temperature was decreased to 250 °C, and 2 mL of the second component was added dropwise over 15 min. Solutions of 0.5 M TOP-Se, 0.25 M TOP-Te, and 0.1 M TOP-Te mixed with 0.4 M TOP-Se were used to make CdSe NRs, CdSe/CdTe HNRs, and CdSe/CdSe_*x*_Te_1−*x*_ HNRs, respectively. Approximately 2/3 of the Cd in the solution was consumed during the growth of seeds, limiting the average overall length of the HNRs to ~25 nm. After 5 additional minutes of growth, the reaction mixture was cooled by removing the heating mantle. The NR/HNR suspensions were cleaned once by precipitation with chloroform and methanol, and redissolved in hexanes. Any insoluble precipitates were discarded, and the NR/HNRs were precipitated again by the addition of chloroform and methanol, dissolved in anhydrous toluene, and stored under N_2_. Recapping with 1-octanethiol followed our previous work on the CdSe/CdTe HNRs^36^. Briefly, 12 mL of 1-octanethiol was added to the flask containing approximately 4 mL of reaction mixture under Ar atmosphere. The reaction vessel was heated to 110 °C, and allowed to cool slowly to 60 °C. After stirring overnight, the HNRs were purified and stored as above.

### Preparation of Polystyrene Microbead-Incorporated TiO_2_ Pastes

Polystyrene (PS) microbead-incorporated TiO_2_ nanoparticle pastes were prepared with P25 nanoparticles (Degussa), ethyl cellulose (EC) as binder, and terpineol as solvent^60^. First, 3 g of P25 powder was added with 0.5 mL of acetic acid and ground for 5 min. Additional grinding for 25 min followed, during which 0.5 mL of ethanol (EtOH) was added every 1 min. Ground P25 powders were dispersed in an excess amount of EtOH. To prepare ethanolic suspension of 200-nm polystyrene beads, aqueous suspensions of PS beads (Alfa Aesar) were dried at 45 °C for 24 h in an oven and obtained solid beads were redispersed in EtOH and sonicated for 1 h. To prepare PS bead-incorporated TiO_2_ pastes, a mixture of ethanolic suspension of P25, ethanolic suspension of PS beads, 10wt.% ethanolic solution of EC, and terpineol was concentrated by evaporating the excess solvents in a 45 °C oil bath with mild stirring. The final composition of the pastes was controlled to be (P25 + PS):EC:solvent = 1.5:1:8 in weight ratio.

### Solar Cell Fabrication

A fluorine-doped tin oxide (FTO) substrate was cleaned and treated by 0.04 M aqueous solution of TiCl_4_ (Sigma-Aldrich, 99.9%) for 30 min at 70 °C, rinsed with deionized water (DIW) and annealed at 450 °C for 30 min to form TiO_2_ blocking layers on the surface^61^. On the TiCl_4_ pre-treated FTO substrate, TiO_2_ paste was spread by the doctor-blade method and annealed for 30 min at 450 °C. Another TiCl_4_ treatment was carried out on the sintered mp-TiO_2_ film using the same conditions as above. The mp-TiO_2_ film was then immersed in a mixture of 3-mercaptopropionic acid (3-MPA, Sigma-Aldrich, ≥99%), acetonitrile (Daejung, Extra Pure), and sulphuric acid (Duksan, Extra Pure) with the volume ratio of 1:9:0.05 for 24 h. The mp-TiO_2_ film functionalized with 3-MPA was then cleaned with acetonitrile and immersed in 3 mM (by Cd content) NR or HNR solution in toluene for 96 h. A ZnS passivation layer was formed on the sensitized film by SILAR, alternately immersing the NR- or HNR-sensitized electrode into a 0.1 M aqueous solution of zinc acetate dihydrate (Zn(CH_3_COOH)_2_ ∙ 2H_2_O, Sigma-Aldrich, ≥98%) and 0.1 M aqueous solution of sodium sulfide nonahydrate (Na_2_S ∙ 9H_2_O, Sigma-Aldrich, ≥98%). Each immersion took 1 min, and the films were rinsed with DIW after each immersion to remove any remaining ions. This cycle was repeated twice to complete the ZnS passivation. To fabricate cuprous sulfide counter electrodes, thoroughly polished brass foil was first etched by hydrochloric acid for 20 min in an 80 °C oven. Etched foil was then sulfurized by adding a polysulfide solution (2 M Na_2_S and 2 M S in DIW) droplet, immediately after which the foil turned black^62^. The NR- or HNR-sensitized mp-TiO_2_ electrode and cuprous sulfide counter electrode were finally assembled into a sandwich-type cell using a binder clip and 60 μm thick scotch tape as a spacer. A solution of 1 M Na_2_S, 1 M S, and 0.2 M KCl in MeOH:DIW = 7:3 solvent was used as the working electrolyte.

### Characterization

Transmission electron microscopy (TEM) was carried out on a JEOL 2100 TEM operating at 200 kV with samples that were prepared by drop-drying a dilute solution of NRs or HNRs in chloroform onto a Cu grid with a thin carbon film (Electron Microscopy Sciences). The UV-vis absorption spectra were collected with an Agilent 8453 photodiode array spectrometer, and photoluminescence spectra were collected with a Horiba Jobin Yvon FluoroMax-3 fluorometer. The morphologies of mp-TiO_2_ electrodes were analysed using a scanning electron microscope (JSM-6360: Hitachi). The photocurrent-voltage (*J*-*V*) curves of SSCs were obtained with a potentiostat (CHI 608C: CH Instrumental Inc., Austin, USA) under AM 1.5 illumination (K3000: McScience, Korea, intensity at 100 mW cm^−2^). An incident photon-to-current conversion efficiency (IPCE) measurement system (K3100: McScience, Korea) was used to obtain IPCE spectra. Electrochemical impedance spectra (EIS) were obtained by a potentiostat with 20 mV sinusoidal perturbation and frequencies ranging from 10^−1^ to 10^5^ Hz.

## Additional Information

**How to cite this article**: Lee, S. *et al.* Integration of CdSe/CdSe_*x*_Te_1–*x*_ Type-II Heterojunction Nanorods into Hierarchically Porous TiO_2_ Electrode for Efficient Solar Energy Conversion. *Sci. Rep.* **5**, 17472; doi: 10.1038/srep17472 (2015).

## Figures and Tables

**Table 1:** Photovoltaic parameters of nanorod (NR)- and heterojunction nanorod (HNR)- sensitized solar cells with different sensitizer structures, compositions and surface ligands.

Sensitizer	*J*_*sc*_ [mA cm^−2^]	*V*_*oc*_ [V]	*FF*	PCE [%]
TOPO-CdSe NR	4.675 ± 0.136	0.579 ± 0.002	0.614 ± 0.002	1.660 ± 0.048
TOPO-CdSe/CdTe HNR	4.856 ± 0.272	0.571 ± 0.004	0.628 ± 0.005	1.739 ± 0.100
TOPO-CdSe/CdSe_0.4_Te_0.6_ HNR	5.377 ± 0.353	0.574 ± 0.002	0.619 ± 0.009	1.911 ± 0.161
OT-CdSe/CdSe_0.4_Te_0.6_ HNR	6.238 ± 0.212	0.579 ± 0.004	0.610 ± 0.012	2.202 ± 0.131

Each parameter is the average with standard deviation from two sets of samples prepared in parallel.

**Table 2:** Photovoltaic parameters of 1-octanethiol (OT) capped CdSe/CdSe_0.4_Te_0.6_ HNR-sensitized solar cells with mesoporous-TiO_2_ (mp-TiO_2_) electrodes derived from the pastes with various polystyrene (PS) microbead contents.

TiO_2_:PS Ratio in Paste (w/w)	*J*_*sc*_[mA cm^−2^]	*V*_*oc*_[V]	*FF*	PCE [%]
No Beads	6.238 ± 0.212	0.579 ± 0.004	0.610 ± 0.012	2.202 ± 0.131
4:1	8.037 ± 0.064	0.582 ± 0.004	0.589 ± 0.011	2.753 ± 0.048
2:1	8.340 ± 0.402	0.595 ± 0.002	0.582 ± 0.011	2.888 ± 0.185
1:1	4.378 ± 0.064	0.605 ± 0.009	0.535 ± 0.014	1.417 ± 0.039
Champion Cell (2:1)	8.624	0.593	0.590	3.019

Each parameter is the average with standard deviation from two sets of samples prepared in parallel.
