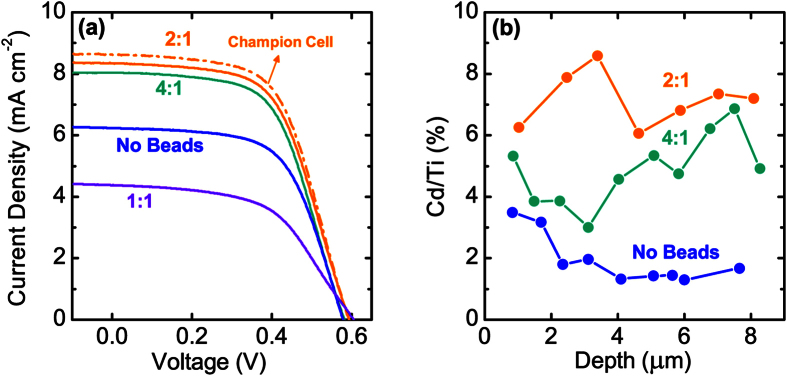# Corrigendum: Integration of CdSe/CdSe_*x*_Te_1−*x*_ Type-II Heterojunction Nanorods into Hierarchically Porous TiO_2_ Electrode for Efficient Solar Energy Conversion

**DOI:** 10.1038/srep26922

**Published:** 2016-05-31

**Authors:** Sangheon Lee, Joseph C. Flanagan, Joonhyeon Kang, Jinhyun Kim, Moonsub Shim, Byungwoo Park

Scientific Reports 5: Article number: 1747210.1038/srep17472; published online: 12 07 2015; updated: 05 31 2016

This Article contained errors.

In the Abstract,

“Additional ~32% enhancement in power conversion efficiency is achieved by introducing percolation channels of large pores in the mesoporous TiO_2_ electrode, which allow 1-D sensitizers to infiltrate the entire depth of electrode.”

now reads:

“Additional ~31% enhancement in power conversion efficiency is achieved by introducing percolation channels of large pores in the mesoporous TiO_2_ electrode, which allow 1-D sensitizers to infiltrate the entire depth of electrode.”

In the Introduction section,

“About 40% enhancement of the PCE is achieved using HNRs compared to the PCE using CdSe nanorods (NRs), which can be attributed to the inherent efficient charge separation across the type-II heterointerface and favorable effects of 1-octanethiol (OT) surface ligands on the TiO_2_-HNR interfacial charge transfer.”

now reads:

“About 33% enhancement of the PCE is achieved using HNRs compared to the PCE using CdSe nanorods (NRs), which can be attributed to the inherent efficient charge separation across the type-II heterointerface and favorable effects of 1-octanethiol (OT) surface ligands on the TiO_2_-HNR interfacial charge transfer.”

In Figure 6b, the x-axis ‘Depth (μm)’ was incorrectly given as ‘Wavelength (nm)’. The correct Figure 6b appears below as Figure 1.

In the Results section under subheading ‘The Effect of Polystyrene Bead-Induced Percolating Pores on the PV Performance’,

“Our simple approach utilizing sacrificial spherical additives results in ~32% additional enhancement in *J*_*sc*_ compared to the OT-CdSe/CdSe_0.4_Te_0.6_ HNR-SSC from the mp-TiO_2_ electrode without polystyrene microbead-induced percolating pores, yielding a 3.02% efficient PV device.”

now reads:

“Our simple approach utilizing sacrificial spherical additives results in ~34% additional enhancement in *J*_*sc*_ compared to the OT-CdSe/CdSe_0.4_Te_0.6_ HNR-SSC from the mp-TiO_2_ electrode without polystyrene microbead-induced percolating pores, yielding a 3.02% efficient PV device.”

These errors have now been corrected in the PDF and HTML versions of the Article.

## Figures and Tables

**Figure 1:**